# Alterations in Surface Electromyography Are Associated with Subjective Masticatory Muscle Pain

**DOI:** 10.1155/2019/6256179

**Published:** 2019-11-22

**Authors:** Davide Pietropaoli, Eleonora Ortu, Mario Giannoni, Ruggero Cattaneo, Alessandra Mummolo, Annalisa Monaco

**Affiliations:** Department of Life Health and Environmental Science, Division of TMD and Orofacial Pain, University of L'Aquila, Building Delta 6, Floor -1, Room #22, San Salvatore Hospital, Zip Code 67100, V.le San Salvatore, L'Aquila, Italy

## Abstract

**Background:**

Tenderness of masseters and temporalis can be considered a relevant tool for diagnosis of myo-type craniofacial pain disorders, but a limit of pain score systems is that they are based on subjective pain perception. Surface electromyography (sEMG) is a noninvasive and reliable tool for recording muscle activity. Therefore, we investigated whether a correlation exists between tenderness on masseters and temporalis, assessed by subjective pain scale, and muscles activity, evaluated by sEMG, in patients with painful temporomandibular disorder (TMD) and concurrent tension-type headache (TTH).

**Methods:**

A cross-sectional study on fifty adult volunteer patients with TMD and TTH, who underwent tenderness protocol according to Diagnostic Criteria for TMD (DC/TMD) guidelines, was conducted followed by sEMG recording of temporalis and masseters. Pearson's correlation was performed to investigate the correlation between muscular activity and subjective pain scores.

**Results:**

An overall moderate correlation between muscle tenderness and sEMG values (*y* = 1 + 1.2 · *x*; *r*^2^ = 0.62; *p* < 0.0001), particularly in the temporalis, was observed. Segregation of data occurred according to tenderness and sEMG values. At the highest pain score, the mean sEMG absolute value was higher at the temporalis than the masseters.

**Conclusions:**

Our study provides evidence that subjective pain perception can be objectively quantified at a magnitude proportional to pain severity. At greater tenderness scores, higher sEMG activity at the level of temporalis could help discriminate clinically prevalent TTH versus prevalent TMD. sEMG confirms to be an accurate tool to reliably objectify the subjective perception of pain. When combined with clinical evaluation and patients' symptoms, sEMG increases diagnostic sensitivity in the field of myo-type craniofacial pain disorders. This trial is registered with NCT02789085.

## 1. Introduction

Pain and muscle dysfunctions are considered keystone symptoms in a temporomandibular joint disorder (TMD) [1] and are often classified as subtypes of a secondary headache disorder [2]. Altered pain perception [3] and dysregulation in pain modulation [4] were recently shown in people suffering from TMD, thus demonstrating lower pain tolerance compared to healthy subjects [3]. Functional brain imaging studies demonstrated increased activation of the somatosensory cortex, anterior cingulate, and prefrontal cortex and decreased thalamic activation in patients with TMD [5]. This neural activation pattern is similar to other chronic pain disorders and may be related to sensitization of pain-producing centers [4, 6]. Clinically, TMD-related pain is often described as myogenous, unilateral, and dull and characterized by variable intensity and duration, ranging from steadily present or intermittent with worsening or improvement periods [7]. The most commonly involved muscles are masseters (MMs) [8–10] and anterior temporalis (ATs) [9, 10]. Palpation-induced pain of these muscles can be considered a relevant tool for differential diagnosis among painful TMD, primary headaches, and bruxism [9]. In addition, palpation-induced pain can be used for assessing subjective pain perception, before and after treatments, using a visual analog scale (VAS) or numerical scale (NS) [11]. However, due to marginal reliability to objective pain quantification, these scales are considered as lacking of scientific rigor [12], but the absence of a “gold-standard” technique for pain assessment promotes its common use in scientific research.

Surface electromyography (sEMG) is a noninvasive technique for recording muscle activity. It is considered a reliable and complementary tool for clinical diagnosis of myogenous TMD and for the study of muscle function [13–15]. In addition, sEMG is considered a reliable tool for investigating the anatomy and physiology of the stomatognathic apparatus [16].

To our knowledge, only a few studies [17] report on the association between palpation-induced pain, or tenderness, and sEMG parameters in individuals with TMD and tension-type headache (TTH).

To this aim, we investigated the correlation between tenderness on masseters and temporalis, assessed by subjective pain scale (NS) using validated criteria for TMD (Research Diagnostic Criteria for TMD: RDC/TMD; and Diagnostic Criteria for TMD: DC/TMD) and objectified muscle activity, evaluated by sEMG, in patients with diagnosis of TMD and concurrent TTH.

## 2. Methods

This cross-sectional study was conducted in accordance with the Declaration of Helsinki. The Committee on Ethics in Science of the University of L'Aquila (L'Aquila, Italy) approved the study, and the written informed consent was obtained from each subject and electronically stored as suggested by our institutional guidelines. All procedures were completed between February and November 2016 at the Division of TMD and Orofacial Pain at University of L'Aquila (L'Aquila, Italy).

### 2.1. Inclusion/Exclusion Criteria

Fifty volunteer patients (39F/11M; mean age 34.8 ± 17.3 years) evaluated with RDC/TMD [18] who fulfilled the following criteria were enrolled in the study: (1) clinical diagnosis of TMD in the last 6 months; (2) diagnosis of TTH based on headache frequency ≥15 days per month for ≥6 months (TTH) [19]; (3) presence of complete permanent dentition, with the exception of the third molars; and (4) normal occlusion. Patients were excluded from the study if they met one or more of the following exclusion criteria: history of local or general trauma; previous diagnosis of systemic diseases, neurological, or psychiatric disorders or muscular diseases; pregnancy; assumption of anti-inflammatory, analgesic, antidepressant, or myorelaxant drugs; fixed or removable prostheses; fixed restorations that affected the occlusal surfaces; and previous or concurrent orthodontic or orthognathic treatment.

In order to confirm TTH, each subject completed a diagnostic headache diary for 4 weeks [20].

Each enrolled subject underwent tenderness protocol followed by sEMG recording, as further specified in the next paragraphs.

### 2.2. Tenderness Protocol

According to the newly evidence-based Diagnostic Criteria for TMD (DC/TMD) [21], pain was evaluated bilaterally on masseter (right = RMM; left = LMM) and anterior temporalis (right = RAT; left = LAT) only, applying 1.0 kg of force for 5 seconds on suggested points [21, 22]. However, since only dichotomous values are present in DC/TMD (pain: yes or no), palpation-induced pain was recorded using a numeric pain scale from 0 to 3, according to RDC/TMD [18].

According to the abovementioned criteria, one point for each palpation area of temporalis (i.e., anterior, middle, and posterior) and masseters (i.e., origin, body, and insertion) was tested, for a total of 12 points (six for each side). The palpation was carried out simultaneously at the left and right sides using both hands. For each muscle, only the highest pain score out of the three measurements was recorded for the analysis.

Finger pressure (1.0 kg) was calibrated twice using a single-hand mechanical algometer (Wagner Instruments, model FPK/FPN, Greenwich, CT, USA) prior to palpation [22–24]. Each hand was calibrated using the same methodology. The areas of palpation were identified as suggested by Ohrbach et al. in the clinical examination protocol for DC/TMD [22].

Palpation was performed with the subjects in a horizontal supine position on a bed with their eyes closed, after 10 minutes of acclimation. Room temperature (21°C) and relative humidity (50%) remained constant. Any external or internal noise sources were controlled. Enrolled patients self-reported the most frequent headache site as occipital, temporal, mixed (occipital plus temporal), or spread.

The same operator (DP) performed the tenderness protocol and registered the induced-pain values in an electronic spreadsheet, reporting the highest pain score for each muscle (RMM, LMM, RAT, and LAT).

### 2.3. sEMG Recording Procedures

According to the literature [15, 25–27], sEMG of masseters (RMM and LMM) and anterior temporalis (RAT and LAT) was recorded simultaneously through surface electromyograph (K7/EMG, Myotronics-Noromed, Inc., Tukwila WA, USA) using disposable silver/silver chloride bipolar surface electrodes (Duotrode; Myotronics-Noromed, Inc., Seattle WA, USA). Before positioning the electrodes, the patient's skin was thoroughly cleaned with alcohol. Electrodes were positioned on the left and right masseter muscles (LMM and RMM) and the left and right anterior temporal muscles (LAT and RAT), as previously described [25]. A template was used to enable repositioning of the electrodes in the same position when the measurements were repeated at different times or if an electrode had to be removed due to malfunction. During the electromyographic examination, the patient was sitting on a chair in the usual conditions with eyes closed. Software for the sEMG K7 (K7 v12.0 Myotronics-Noromed, Inc., Tukwila WA, USA) was set up in order to record RMM, LMM, RAT, and LAT only. The ground electrode, which was common to all channels and larger than the others for a proper contact with the skin, was positioned on the subject's forehead to ensure a common reference to the differential input of the amplifier [15]. Electrical signals were amplified, recorded, and digitized with the K7 clinical software package. The root mean square (RMS) values (in *μ*V) were used as indices of the signal amplitude [16].

Three consecutive sEMG rest position tracks, with a duration of 15 seconds each, were acquired, and any trace with interposing phasic event, such as swallowing, voluntary movement, or clenching, was discarded. The sEMG recording procedure was subsequent to the palpation, 5 minutes after placing the electrodes. The recording procedure was carried out in the same room where the tenderness protocol was performed.

Procedure for sEMG registration was performed by one examiner (AM) in a blinded fashion. For each studied muscle, the average between the three recordings, which was generated by the software and expressed as microvolts (mV), was reported in a spreadsheet for differential statistics.

### 2.4. Statistical Analysis

Parametric approach was used for a differential statistic since collected data revealed normal distribution with the Shapiro–Wilk test. Relation between sEMG and palpation-induced pain values was evaluated by Pearson's correlation.

Level of significance was set at *p* < 0.05. The R software was used for statistical analysis [28]. Moreover, plots and heatmaps were generated with R packages “ggplot2” and “pheatmap.”

## 3. Results

**Figure 1:**
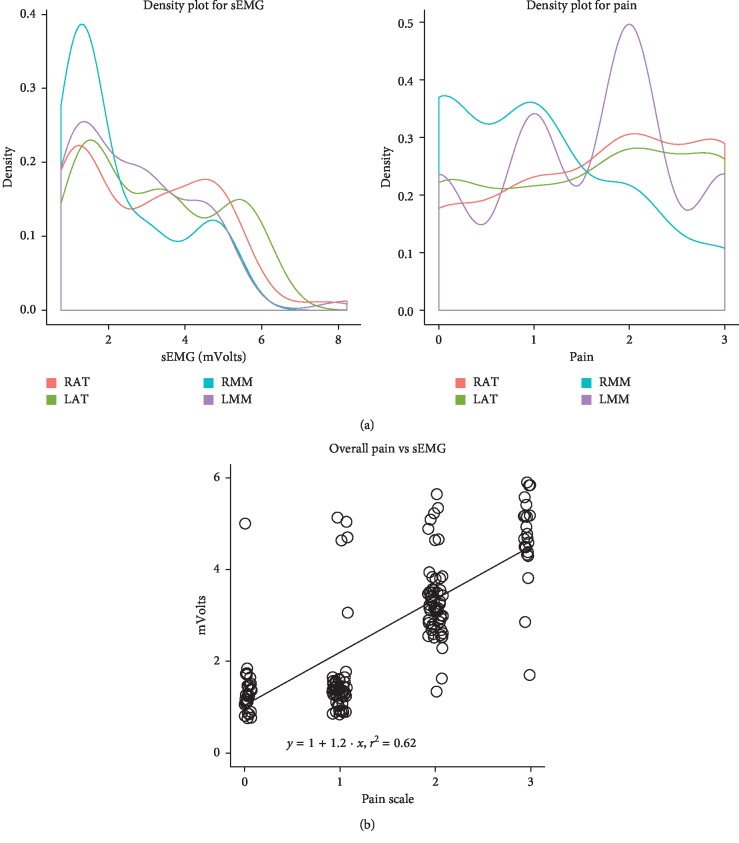
(a) Density plot for sEMG (mVolts) and pain (scale 0–3) at the level of right anterior temporalis (RAT), left anterior temporalis (LAT), right masseter muscle (RMM), and left masseter muscle (LMM). (b) Overall moderate correlation between muscle tenderness and sEMG values (*y* = 1 + 1.2*x*; *r*^2^ = 0.62; *p* < 0.0001). Segregation of data occurs according to tenderness and sEMG values, with lower pain scores (0-1) associated with lower sEMG values (<2 mV) and higher pain scores (2-3) associated with higher sEMG values (>2 mV).

Results from statistical analysis shown an overall moderate correlation between muscle tenderness and sEMG values (*y* = 1 + 1.2 · *x*; *r*^2^ = 0.62; *p* < 0.0001) (Figure 1).

**Figure 2:**
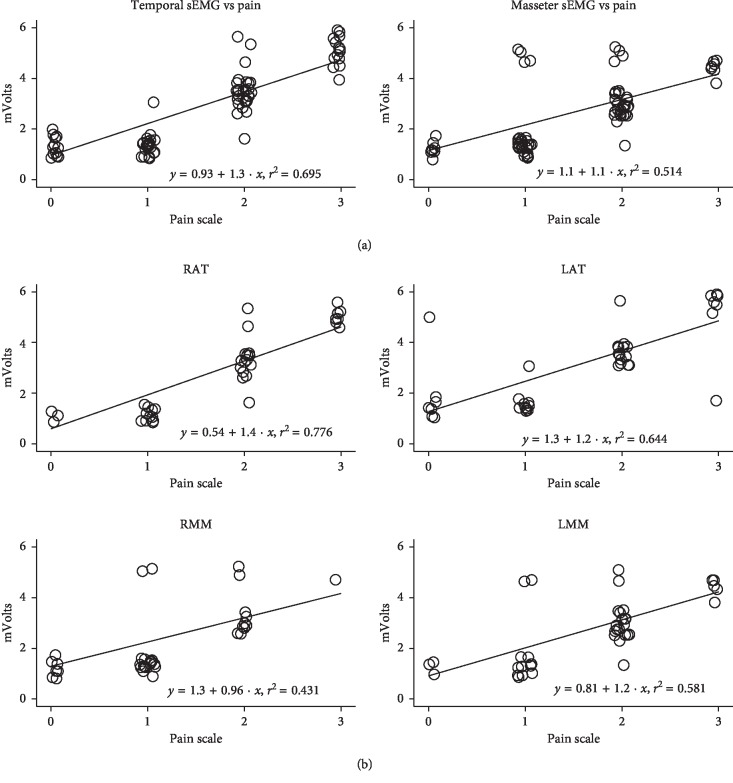
(a). Moderate correlation between electric values and palpation-induced pain of temporalis (*y* = 0.93 + 1.3*x*; *r*^2^ = 0.695; *p* < 0.0001) and masseters (*y* = 1.1 + 1.1*x*; *r*^2^ = 0.514; *p*=0.0062). At the highest pain score (=3), the mean sEMG absolute value (expressed in mV ± SD) is significantly higher at the ATs compared to the MMs (5.16 ± 0.47 mV vs 4.73 ± 0.30 mV, respectively; *p* < 0.001). (b) Pearson's analysis of single muscle sEMG findings and tenderness: strong correlation for RAT (*y* = 0.54 + 1.4*x*; *r*^2^ = 0.776; *p* < 0.0001); moderate correlation for LAT (*y* = 1.3 + 1.2 · *x*; *r*^2^ = 0.644; *p*=0.0011); moderate-low correlation for RMM (*y* = 1.3 + 0.96·*x*; *r*^2^ = 0.431; *p*=0.0032); moderate correlation for LMM (*y* = 0.81 + 1.2 · *x*; *r*^2^ = 0.581—*p*=0.0043).

The investigation of muscle types showed a moderate correlation between electric values and palpation-induced pain of both temporalis and masseters (temporalis: *y* = 0.93 + 1.3*x*; *r*^2^ = 0.695; *p* < 0.0001*—*masseters: *y* = 1.1 + 1.1*x*; *r*^2^ = 0.514; *p*=0.0062) (Figure 2(a)).

Pearson's analysis of single muscle sEMG findings and palpation-induced pain revealed a strong correlation for RAT (*y* = 0.54 + 1.4*x*; *r*^2^ = 0.776; *p* < 0.0001), a moderate correlation for LAT (*y* = 1.3 + 1.2*x*; *r*^2^ = 0.644; *p*=0.0011), a moderate-low correlation for RMM (*y* = 1.3 + 0.96*x*; *r*^2^ = 0.431; *p*=0.0032), and a moderate correlation for LMM (*y* = 0.81 + 1.2*x*; *r*^2^ = 0.581; *p*=0.0043) (Figure 2(b)).

**Figure 3:**
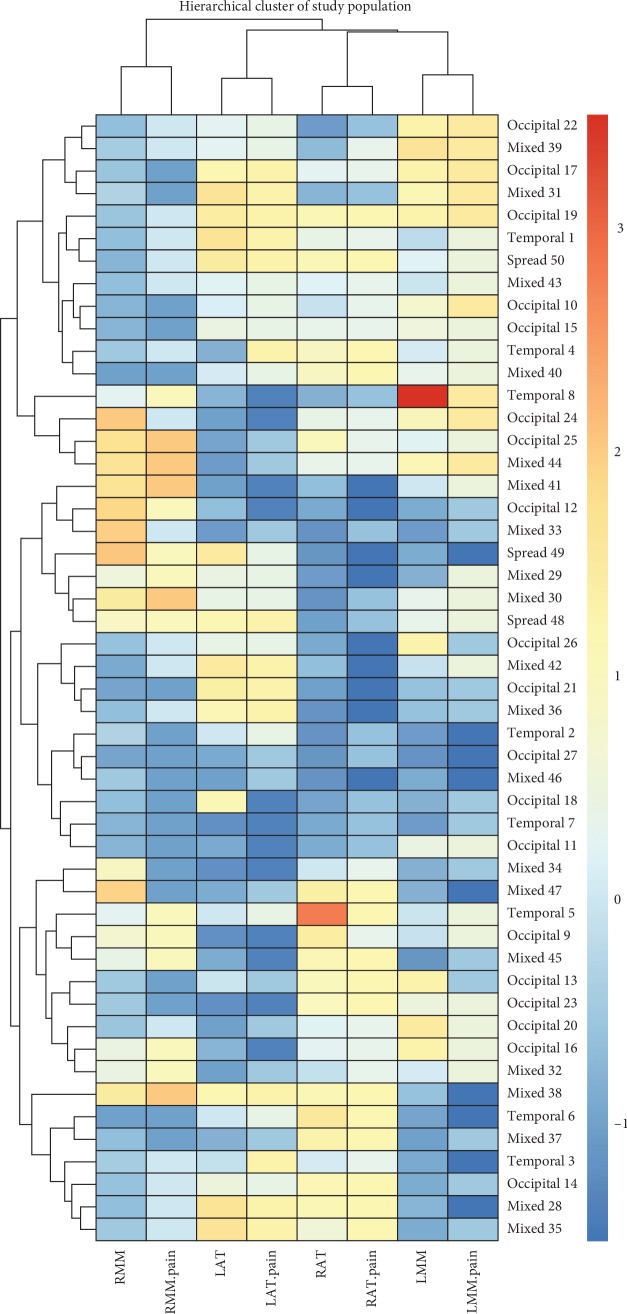
Hierarchical cluster analysis. Single muscle tenderness and relative muscles were paired by the Euclidean correlation (columns).

The hierarchical cluster analysis paired muscles and relative pain perception using an euclidean correlation (columns) Figure 3.

When considering the sEMG findings as a whole (Figure 1(b)), segregation of data occurs according to tenderness and sEMG values, with lower pain scores (0-1) associated with lower sEMG values (<2 mV) and higher pain scores (2-3) associated with higher sEMG values (>2 mV) (Table 1).

Mean sEMG absolute values (expressed in mV ± SD) at pain scores between 0 and 2 did not significantly differ between the ATs and MMs groups. Indeed, at the highest pain score (=3), the mean sEMG absolute value was 5.16 ± 0.47 mV and 4.73 ± 0.30 mV at the ATs and the MMs, respectively (*p* < 0.001) (Figure 2(a)).

## 4. Discussion

Our study demonstrates three important findings. First, sEMG findings in individuals with TMD and concurrent TTH correlate with temporal and masseter tenderness, and this correlation is particularly strong at the level of ATs. Second, sEMG confirms to be an accurate tool to reliably objectify the subjective perception of pain. Third, at greater tenderness scores, higher sEMG activity at the level of ATs compared to MMs could help discriminate clinically prevalent TTH vs prevalent myo-type TMD (Figure 2(a)).

With reference to the first point, tenderness at the ATs and MMs has been documented in individuals suffering from TMD and TTH [10]. Interestingly, the previous literature reported that subjects with TMD and concurrent TTH more frequently showed positive trigger points at the ATs, whereas tenderness and active trigger points were more typically observed at the MM level in the presence of isolated TMD without TTH [29]. Our findings confirm the observation of major involvement of ATs in the TMD-TTH phenotype both in terms of clinical burden and instrumental evidence. In this perspective, our study provides evidence that subjective pain perception can be objectively quantified at a magnitude proportional to pain severity.

According to this, segregation of data occurred based on tenderness and sEMG values, with a direct relationship between the two parameters (Figure 1(b)). The previous literature reported on increased sEMG at rest at the level of ATs [30] and trapezius [31] in patients with TTH, which was associated with the magnitude of pain perception. Increasing evidence also shows that sEMG is useful in the differential diagnosis between healthy subjects and TMD patients [13, 14, 32]. With reference to individuals with TMD, there is to date no agreement on the normal values of basal muscle activity measured with sEMG at the level of ATs and MMs, with some authors suggesting increased values [17, 33] and others no difference [34, 35], in comparison to otherwise healthy subjects. These studies, however, do not take into account the possible concomitant presence of TTH in the examined subjects: therefore, a misclassification might have occurred by excluding individuals with TMD and concomitant TTH, thus including only low-tenderness, low-sEMG TMD individuals.

Comorbidity of TMD and TTH is well documented in the literature [36, 38] and goes beyond any fortuitous occurrence. Possible pathogenetic mechanisms underlying TMD and TTH association include central and peripheral sensitization [39–41]. According to Conti et al., these two conditions share overlapping pathogenetic and clinical features, including response to treatments, thus making it hard to effectively and distinctly classify one or the other in terms of scoring systems routinely used in neurology (International Classification of Headache Disorders, ICHD-3) or dentistry (RDC/DC) [42]. In this perspective, sEMG represents a reliable tool in combination with clinical evaluation and patients' symptoms, in order to increase diagnostic sensitivity in the field of myo-type craniofacial pain disorders.

Our results also demonstrated significantly higher absolute values of sEMG findings at greater tenderness scores at the level of ATs compared to MMs. Although little differences in mV amplitude between ATs and MMs may occur, as expression of different skin impedance at the two sites [43], and although absolute sEMG values may have a secondary significance when interpreting similar results, the evidence of high sEMG values at the ATs, in association with high tenderness scores at the same level, may identify the presence of clinically prevalent TTH.

This study has some limitations that deserve to be mentioned. First, the lack of control group of healthy subjects since it is a cross-sectional study; data were extrapolated by using software as RMS and finally the sEMG measurement was made only after muscle palpation, and these data indicate only a correlation between subjective pain and sEMG values, thus the generalizability deserves caution.

## 5. Conclusions

In conclusion, pure myo-facial TMD without TTH may be characterized by the lack of such a manifest activation of ATs. The clinical meaningfulness of these observations needs to be evaluated in the light of further knowledge, particularly studies looking at the therapeutic response to different treatment approaches.

## Figures and Tables

**Table 1:** Mean and standard deviation (SD) of sEMG and subjective pain score relative to investigated muscles of the enrolled patients.

		Occipital	Spread	Temporal	Mixed	*p*
	*n*	8	20	19	3	
sEMG	RAT (mean (SD))	2.99 (1.57)	2.93 (1.68)	2.77 (1.40)	2.27 (2.19)	0.905
	LAT (mean (SD))	3.70 (1.90)	3.21 (1.84)	2.84 (1.63)	5.46 (0.26)	0.104
	RMM (mean (SD))	1.65 (0.65)	2.65 (1.52)	2.02 (1.42)	3.25 (2.07)	0.185
	LMM (mean (SD))	2.37 (1.65)	2.35 (1.28)	3.39 (1.48)	2.44 (1.05)	0.117

Subjective pain	RAT.pain (mean (SD))	2.00 (0.93)	1.70 (1.22)	1.68 (1.00)	1.33 (1.53)	0.826
	LAT.pain (mean (SD))	1.88 (1.25)	1.70 (1.08)	1.37 (1.16)	3.00 (0.00)	0.132
	RMM.pain (mean (SD))	0.88 (0.83)	1.60 (1.14)	0.95 (1.13)	2.00 (1.00)	0.133
	LMM.pain (mean (SD))	1.38 (1.30)	1.45 (1.00)	2.16 (0.96)	1.33 (1.15)	0.129

sEMG: surface electromyography; RAT: right anterior temporal muscle; LAT: left anterior temporal muscle; RMM: right masseter muscle; LMM: left masseter muscle. Stratification according to headache is also shown.

## Data Availability

Data will be shared at request according to the institutional policy.
